# Perinatal outcomes and growth discordance of triplet pregnancies based on chorionicity: a retrospective cohort study

**DOI:** 10.1186/s12884-024-06599-5

**Published:** 2024-05-28

**Authors:** Qing Hu, Zhaomin Zeng, Hongyan Liu, Hua Liao, Tingting Xu, Haiyan Yu

**Affiliations:** 1 Department of Obstetrics and Gynecology, West China Second University Hospital, Sichuan University, No. 20, 3 Section, South Renmin Road, Chengdu, Sichuan 610041 China; 2 Laboratory of Birth Defects and Related Diseases of Women and Children (Sichuan University), Ministry of Education, Chengdu, China

**Keywords:** Trichorionic triplets, Dichorionic triplets, Monochorionic triplets, Perinatal outcomes, Birth weight discordance, Triplet pregnancy

## Abstract

**Background:**

The worldwide occurrence of triplet pregnancy is estimated to be 0.093%, with a natural incidence of approximately 1 in 8000. This study aims to analyze the neonatal health status and birth weight discordance (BWD) of triplets based on chorionicity from birth until discharge.

**Methods:**

This was a retrospective study. We reviewed a total of 136 triplet pregnancies at our tertiary hospital between January 1, 2001, and December 31, 2021. Maternal and neonatal outcomes, inter-triplet BWD, neonatal morbidity, and mortality were analyzed.

**Results:**

Among all cases, the rates of intrauterine death, neonatal death, and perinatal death were 10.29, 13.07, and 24.26%, respectively. Thirty-seven of the cases resulted in fetal loss, including 13 with fetal anomalies. The maternal complications and neonatal outcomes of the 99 triplet pregnancies without fetal loss were compared across different chorionicities, including a dichorionic (DC) group (41 cases), trichorionic (TC) group (37 cases), and monochorionic (MC) group (21 cases). Neonatal hypoproteinemia (*P* < 0.001), hyperbilirubinemia (*P* < 0.019), and anemia (*P* < 0.003) exhibited significant differences according to chorionicity, as did the distribution of BWD (*P* < 0.001). More than half of the cases in the DC and TC groups had a BWD < 15%, while those in the MC group had a BWD < 50% (47.6%). TC pregnancy decreased the risk of neonatal anemia (adjusted odds ratio [AOR] = 0.084) and need for blood transfusion therapy after birth (AOR = 0.119). In contrast, a BWD > 25% increased the risk of neonatal anemia (AOR = 10.135) and need for blood transfusion after birth (AOR = 7.127). TC pregnancy, MCDA or MCTA, and BWD > 25% increased neonatal hypoproteinemia, with AORs of 4.629, 5.123, and 5.343, respectively.

**Conclusions:**

The BWD differed significantly according to chorionicity. Additionally, TC pregnancies reduced the risk of neonatal anemia and need for blood transfusion, but increased the risk of neonatal hypoproteinemia. In contrast, the BWD between the largest and smallest triplets increased the risk of neonatal anemia and the need for blood transfusion. TC pregnancy, MCDA or MCTA, and BWD > 25% increased the risks of neonatal hypoproteinemia. However, due to the limited number of triplet pregnancies, further exploration of the underlying mechanism is warranted.

## Background

The occurrence of triplet pregnancy has been reported to be 0.093%, with a natural incidence of 1/8000 [1]. Triplet pregnancy is the most common type of high-order multiple gestations and is most likely to occur after infertility treatment [2]. Indeed, 0.9% of pregnancies conceived by assisted reproductive technology (ART) are higher-order multifetal pregnancies [3]. Furthermore, the latest data in the United States estimated that 1 in 1880 pregnancies is a triplet pregnancy [4].

Triplet pregnancies increase the risk of miscarriage and preterm birth [5, 6]. Moreover, the occurrence of hypertensive complications has been reported to be approximately three times higher than that in singleton pregnancies and 1.5 times higher than that in twin pregnancies [6, 7]. Reported neonatal outcomes are also somewhat pessimistic, with a preterm delivery (< 32 weeks) rate of approximately 37% in triplet pregnancy, which is 3.5 times higher than that of twins and 31 times higher than that of singleton pregnancies [7]. One study also demonstrated that, compared to twins, triplets have a four-fold increased risk for birth after less than 29 weeks of gestation; furthermore, the risk of death or neurodevelopmental impairment is also high in low-birth-weight infants from triplet pregnancies when compared to twins and singletons [8]. Thus, prematurely born triplet neonates exhibit an associated increase in short- and long-term sequelae. Data from a meta-analysis of 43,653 triplets presented a mean birth age of 32.3 gestational weeks [1], including 98.3% of neonates born before 37 gestational weeks, with 2.8% exhibiting cerebral palsy and with an overall infant mortality rate of 5.25% [6]. Twin–twin transfusion syndrome (TTTS), a potential cause of in-utero demise, and medically indicated preterm delivery occur in 10%–15% of monochorionic twins, as well as in dichorionic triamniotic (DCTA) and monochorionic triamniotic (MCTA) triplets, in which the prevalence is currently unknown [9]. Reports have shown that among confirmed cases of triplets at 5–6 weeks of gestation, more than 50% have a spontaneous reduction of one or more embryos before 12 weeks. The risk of spontaneous loss of the pregnancy before 24 weeks is 15–18% for triplets and 8% for twins. Moreover, the intrauterine fetal demise of one or more triplets, which is higher than that for twin or singleton pregnancies, is estimated to be 1–3% or more, depending on gestational age and chorionicity [10]. Taken together, all of these factors yield a heavy healthcare cost burden.

Triplet pregnancies can present in different combinations of mono-, di-, or tri-chorionicity, as well as mono, di-, or tri-amnionicity. The incidence of dichorionic triplet pregnancies is 44% in naturally conceived triplets and 1.24% after ART [10, 11]. Fennessy et al. [12] reported that spontaneous triplet pregnancies were composed of 43% trichorionic triamniotic (TCTA) triplets, 39% DCTA triplets, 14% MCTA triplets, and 4% dichorionic diamniotic (DCDA) triplets, while Curado et al. [13] reported 77.3% TCTA, 19.01% DCTA, and 3.64% MCTA triplets. Previous studies have proposed that chorionicity is a major determinant of perinatal and maternal outcomes in triplet pregnancies [2]. DCTA has been reported to carry a 3.3 higher risk of perinatal death than TCTA pregnancies [14]. Furthermore, the risk of neurological morbidity was reported higher in DCTA than in TCTA triplets (OR = 5.4) [13]. MCTA triplets have also been reported to have a neurological morbidity prevalence of 2.7%, a respiratory morbidity prevalence of 25.3%, an infectious morbidity prevalence of 3.3%, and a composite morbidity prevalence of 25.3% [13].

Studies have shown that monochorionic placentation of a pair or trio in triamniotic triplet pregnancy is associated with a significantly increased stillbirth risk due to greater size discordance [14]. An article published in 2003 also stated that a birth weight discordance (BWD) greater than or equal to 15% (same sex) or 30% (different sex) confers the greatest risk of adverse perinatal outcomes in the absence of abruption in twin pregnancies [15].

To the best of our knowledge, few previous studies have focused on the analysis of the neonatal status of different chorionic triplets and BWD from birth to hospital discharge. Herein, we performed a retrospective cohort study to analyze the neonatal health status and BWD based on chorionicity from birth until discharge. We also analyzed maternal characteristics according to chorionicity.

## Materials and methods

A retrospective cohort study was conducted to investigate the perinatal outcomes and BWD of triplet pregnancies according to chorionicity. The patients were recruited between January 1, 2001, and December 31, 2021, at a tertiary hospital in West China. Cases of triplet pregnancies that were not delivered at our hospital or lacked complete medical records were excluded. Approval was obtained from the Institutional Review Board of West China Second University Hospital. All data were abstracted from medical records. The patients were categorized into two groups: triplet pregnancies with fetal loss and triplet pregnancies without fetal loss. Further subgrouping was performed according to chorionicity.

After confirming fetal malformations and chromosomal abnormalities, the pregnant woman and her partner were extensively counseled by the multidisciplinary team regarding the treatment and prognosis of the affected fetus. Then, selective reduction was determined according to the couple’s choice and the chorionicity. For monochorionic fetuses, umbilical cord bipolar coagulation or intrafetal laser therapy was used, while intracardiac injection of potassium chloride was used for dichorionic fetuses.

The gestational age of included cases was determined based on the patient’s last menstrual period in women with regular cycles or on the first-trimester ultrasound scan. The chorionicity and amnionicity of the triplet pregnancies were detected by an ultrasound specialist in the first trimester. The chorionicity was validated using the following indicators: the number of placental masses, the presence of amniotic membrane(s) and membrane thickness, and the lambda or T-sign [16]. The lambda sign is the triangular projection of tissue extending up to the base of the intertwin membrane; the presence of the lambda sign indicates the presence of two placentas, while the presence of two lambda signs indicates trichorionicity. In contrast, a thin intermembrane with a base that meets the placenta at a 90º angle, known as the “T sign,” is diagnostic of monochorionicity [10]. Chorionicity and amnionicity were confirmed by postnatal pathology.

The baseline data collected included maternal age, height, weight before pregnancy, body mass index (BMI), gravity, parity, abortion times, hemoglobin (HGB) before birth and platelet levels before birth, and gestational week at birth. The rate of amniocentesis, vaginal bleeding in the first or second trimester, hypertension, pregnancy mode, and cesarean section in maternal medical cases were abstracted manually.

Intrauterine death (IUD) was defined as the death of at least one fetus from 20 weeks gestation onwards, neonatal death (NND) was defined as the death of at least one of the newborns within 28 days after birth, and perinatal death (PND) was defined as IUD plus NND [12]. Triplet pregnancies with three fetuses born alive were defined as being without fetal loss. Triplet pregnancies with one, two, or zero fetuses born alive were defined as being with fetal loss.

Neonatal outcomes included gestational age at birth, survival rate, rate of transfer to the neonatal intensive care unit (NICU), and Apgar score at 5 min. The associated rate of malformation, asphyxia, pneumonia, apnea, and bronchopulmonary dysplasia were recorded. The BWD was calculated using the following formula as previously described [14]: ([birth-weight largest fetus] – [birth-weight smallest fetus])/birth-weight largest fetus × 100%.

The collected data encompassed maternal baseline characteristics, complications, chorionicity, gestational age at delivery, birth weight (BW), admission to the NICU, therapy in the NICU, neonatal conditions, and complications after birth.

The data were compiled and examined using the R programming language (version 4.1.1; R Foundation for Statistical Computing, Vienna, Austria). Descriptive statistics are presented as the median and interquartile range (IQR) and percentages for enumerated data. The data were divided into three groups for comparison according to chorionicity. Statistical analyses were conducted using the Mann–Whitney U test. Continuous data were analyzed using one-way ANOVA or the Kruskal–Wallis H Test as appropriate, while the chi-squared, Fisher’s exact, or association tests were used for proportions. A multivariate binary logistic regression analysis was conducted to investigate the BWD effect on the perinatal outcome of triplet pregnancy according to chorionicity. *P* < 0.05 was considered to indicate statistical significance.

## Results

**Fig. 1:**
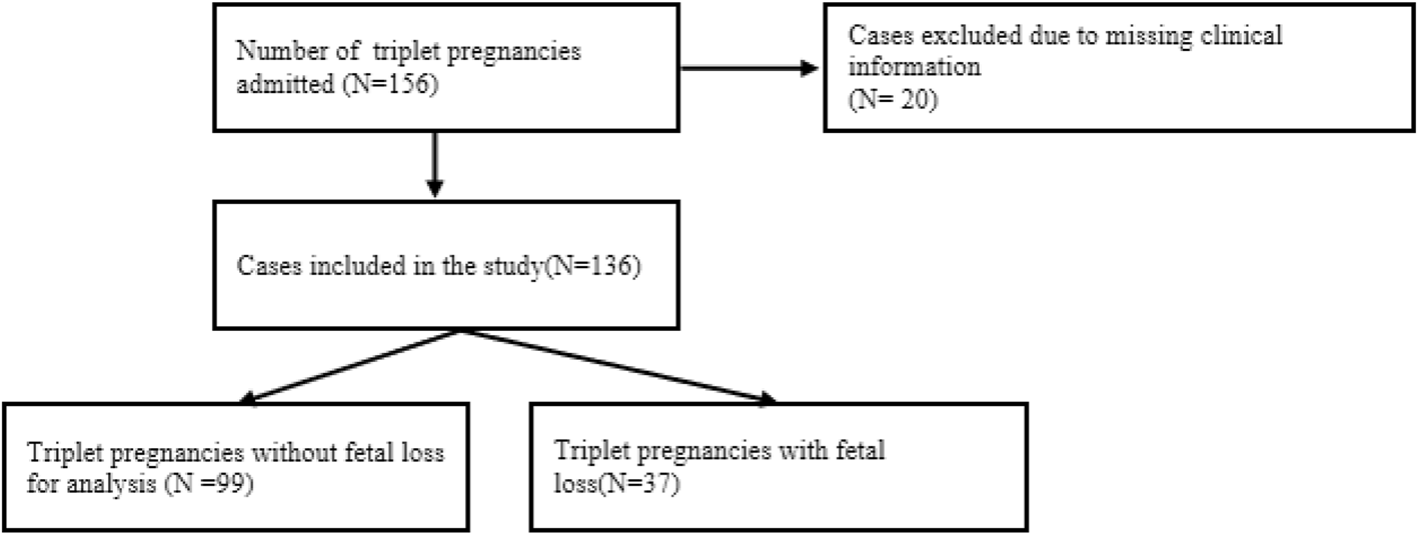
Flowchart of the study

**Fig. 2:**
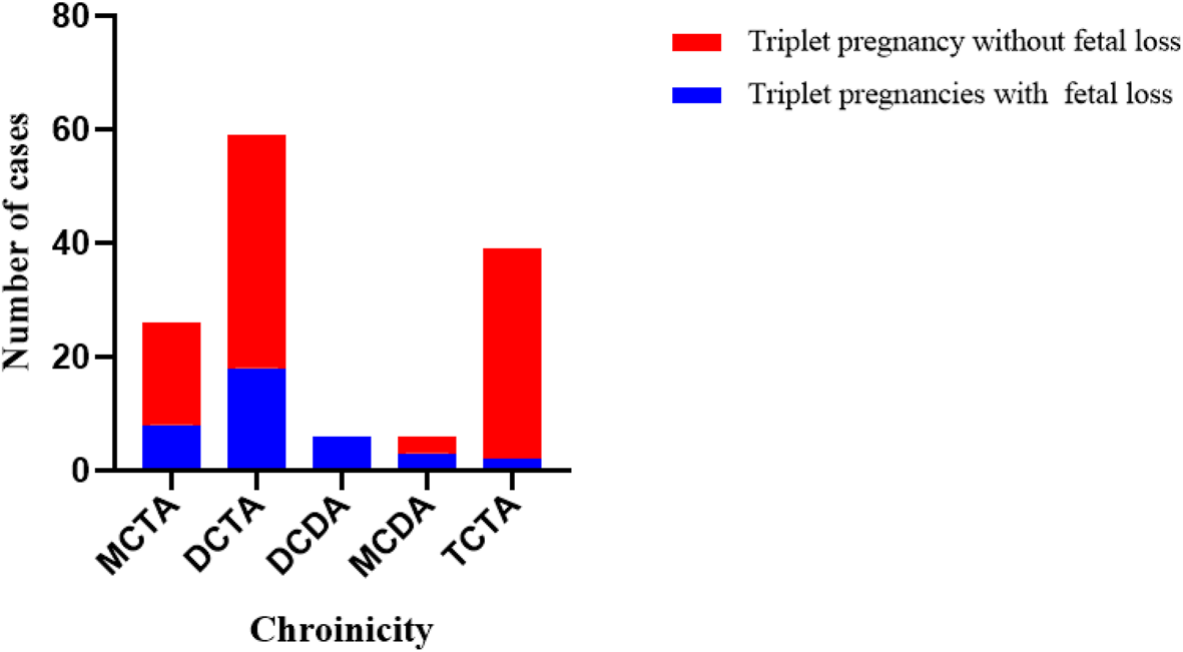
Distribution of chorionicity in 136 triplet pregnancies

During the study period, 156 triplet pregnancies were delivered at our hospital, but 20 cases were not included due to missing clinical information. Therefore, 136 triplet pregnancies with complete records were finally included in this study, with an incidence of triplet pregnancy of 0.094% (156/165985). The detailed research process is depicted in Fig. 1, and the distribution of chorionicity is displayed in Fig. 2. In this study, 19.12% of the cases were MCTA, 43.38% were DCTA, 4.41% were DCDA, 4.41% were MCDA, and 28.68% were TCTA.

Maternal characteristics such as height, weight before pregnancy, gravidity, parity, abortion times, and rate of complications were compared among different chorionicity groups. Detailed information is shown in Tables 1 and 2. The results demonstrated differences in height, weight before pregnancy, and pregnancy mode according to chorionicity (*P* < 0.05). DCTA pregnancies exhibited a taller height and a higher HGB before birth than other triplets, while TCTA pregnancies were associated with a higher BMI at birth. More than 50% of DC and MC triplets were conceived naturally, while the DCTA pregnancies had the highest rate of IVF-ET.

**Table 1:** Demographic characteristics of included triplet pregnancies

	**DCTA**	**TCTA**	**MCDA or MCTA**	***P***
N	64	39	33	
Maternal age (mean (SD))^c^	30.35 (4.89)	28.92 (6.68)	28.27 (4.98)	0.172
Height (mean (SD))^c^	160.92 (4.61)	158.77 (4.10)	159.00 (3.89)	**0.024**
Pre-weight (median [IQR])^d^	55.10 [50.00, 60.00]	55.00 [50.00, 60.75]	53.00 [49.00, 55.00]	**0.048**
Pre-BMI (median [IQR])^d^	21.51 [19.55, 23.03]	22.07 [19.93, 24.83]	21.30 [18.89, 22.03]	0.077
Maternal weight before delivery (mean (SD))^c^	71.46 (7.78)	74.16 (10.36)	69.61 (8.25)	0.084
BMI before delivery (mean (SD))^c^	27.60 (2.94)	29.39 (3.69)	27.54 (3.18)	0.015
Gravidity (mean (SD))^c^	2.34 (1.35)	2.23 (1.55)	2.09 (1.23)	0.692
Parity (mean (SD))^c^	0.36 (0.52)	0.18 (0.51)	0.30 (0.59)	0.251
Abortion times (mean (SD))^c^	0.87 (1.21)	0.97 (1.16)	0.82 (0.92)	0.832
HGB_before birth (mean (SD))^c^	116.79 (12.78)	111.66 (15.57)	109.06 (16.14)	**0.042**
PLT_before_birth (mean (SD))^c^	162.02 (47.39)	144.82 (54.37)	166.12 (58.26)	0.177
Uterine_leiomyomas (%)^a^	2 (3.1)	0 (0.0)	1 (3.0)	0.607
PCOS (%)^a^	2 (3.1)	5 (12.8)	1 (3.0)	0.119
Thalassemia (%)^a^	1 (1.6)	1 (2.6)	0 (0.0)	1
Pregnancy_mode (%)^a^				** < 0.001**
Natural	34 (53.1)	17 (43.6)	29 (87.9)	
Ovulation_induction	4 (6.2)	9 (23.1)	0 (0.0)	
IVF-ET	26 (40.6)	13 (33.3)	4 (12.1)	
Amniocentesis (%)^b^	16 (25.0)	4 (10.3)	6 (18.2)	0.18

*VB* Vaginal bleeding, *CS* Cesarean section, *IVF-ET* In vitro fertilization-embryo transfer, *SD* Standard deviation, *IQR* Interquartile range, *HGB* Hemoglobin, *PLT* Platelet, *PCOS* Polycystic ovarian syndrome

^a^Fisher’s exact test

^b^Chi-squared test

^c^One-way ANOVA

^d^Mann–Whitney test

**Table 2:** Maternal pregnancy complications among the included triplet pregnancies

	**DCTA**	**TCTA**	**MCDA or MCTA**	***P***
N	64	39	33	
VB at first or second trimester (%)^b^	17 (27.0)	10 (25.6)	3 (9.1)	0.112
Polyhydramnios (%)^a^	5 (7.8)	4 (10.3)	8 (24.2)	0.068
Hypertension (%)^b^	8 (12.5)	7 (17.9)	10 (30.3)	0.248
ICP (%)^b^	18 (28.1)	16 (41.0)	8 (24.2)	0.248
GDM (%)^b^	12 (18.8)	5 (12.8)	4 (12.1)	0.6
HELLP (%)^a^	0 (0.0)	0 (0.0)	0 (0.0)	1
PROM (%)^b^	18 (28.1)	9 (23.1)	10 (30.3)	0.77
Placenta_previa (%)^a^	1 (1.6)	4 (10.3)	1 (3.0)	0.103
TTTS (%)^a^	3 (4.7)	0 (0.0)	3 (9.1)	0.132
Placenta_accreta (%)^a^	4 (6.2)	1 (2.6)	0 (0.0)	0.443
Rate of CS (%)^b^	49 (76.6)	34 (87.2)	23 (76.7)	0.385
Gestational week at birth (mean (SD))^c^	32.44 (4.98)	33.16 (3.00)	32.66 (3.72)	0.706

*VB* Vaginal bleeding, *ICP* Intrahepatic cholestasis during pregnancy, *GDM* Gestational diabetes mellitus, *PROM* Premature rupture of membranes, *TTTS* Twin-to-twin transfusion syndrome, *CS* Cesarean section, *HELLP* Hemolysis, elevated liver enzymes, and low platelets

^a^Fisher’s exact test

^b^Chi-squared test

^c^One-way ANOVA

The rates of IUD, NND, and PND were 10.29% (42/408), 13.07% (57/408), and 24.26%, respectively, in 136 triplet pregnancies. To avoid statistical bias, the 136 triplet pregnancies were divided into two groups: triplet pregnancies with (37 cases) and without (99 cases) fetal loss, according to whether all three fetuses were born alive.

### Perinatal outcomes in triplet pregnancies with fetal loss

Among the 37 triplet pregnancies with fetal loss, 13 cases were diagnosed with anomalies, with the following incidences: 6.62% for acardiac fetus, 0.74%, for syndactyly of the left hand, 1.47% for lymphatic cystoma, 1.47% for conjoined twin, 0.74% for omphalocele combined with cystocele, and 1.47% for hydrocrania, cleft lip and palate, anencephaly, and hydramnios. Among the pregnancies impaired by an acardiac fetus, the distribution of chorionicity and incidence upon chorionicity were as follows: 3 MCTA, 3 MCDA, 2 DCDA, and 1 DCTA.

IUD occurred in 19 cases/42 fetuses, and NND occurred in 6 cases/14 fetuses; selective feticide was performed in 12 cases. Miscarriage occurred in 5 cases, vaginal delivery in 5 cases, cesarean section in 22 cases, and termination of pregnancy in 5 cases. Selective feticide was implemented in 11 cases, including seven and four cases of reduction to twin and singleton pregnancies, respectively. The mean birth weeks and BW in cases reduced to twin were 33.65 weeks and 1731.43 g, while those in cases reduced to singleton were 37.50 weeks and 3045.00 g, respectively. Additionally, there were 19 triplet pregnancies with intrauterine deaths, with 3 fetal deaths in 10 cases, 2 fetal deaths in 3 cases, and 1 fetal death in 6 cases. Among all surviving fetuses, one neonate was complicated with brain damage. The detailed information on triplet pregnancies with fetal loss is shown in Table 3, along with the chorionicity distribution of the 37 cases.

**Table 3:** Triplet pregnancies with fetal loss

Chorionicity	Delivery age and mode	T1	T2	T3
MCTA	35 + 5, CS	2470 g, M, syndactylia of left hand	2780 g, M	500 g, acardiac
DCTA	36 + 6, CS	2630 g, F	2540 g, F	IUD
DCTA	22 + 3, miscarriage	IUD	IUD	IUD
DCTA	38 + 5, CS	3390 g	IUD, MC, lymphatic cystoma	IUD, MC
MCTA	21 + 3, TOP	Acardiac, SF	Hydramnios	Hydramnios
DCTA	37 + 6, CS	3080 g, F	IUD	IUD
DCTA	36 + 5, CS	2300 g, M	2720 g, M	SF
DCTA	36 + 1,CS	2120 g, M	2140 g, M	IUD
DCDA	37, CS	3270, F	Conjoined twin, lymphatic cystoma, SF	Conjoined twin, SF
DCDA	37 + 4, CS	2769, F	Conjoined twin, SF	Conjoined twin, SF
DCDA	37 + 6, VD	3640 g, F	IUD, MCDA withT3	Omphalocele, cystocele, SF
DCDA	14 + 5, TOP	IUD	IUD	IUD
MCDA	37 + 4, CS	2510 g, F	Acardiac, SF	Acardiac, SF
DCDA	37 + 2, CS	2290 g, M	2000 g, F	Acardiac, growth stopped
DCTA	32 + 2, CS	2090 g, M	1759 g, F	550 g, acardiac
MCTA	30, CS	1400 g, F	1190 g, F	Acardiac, SF
MCDA	33, CS	1880 g, M	1630 g, M	Acardiac, SF
DCTA	31 + 6, CS	1480 g, F, hydrocrania	IUD	1600 g, M
DCDA	33 + 1, CS	1700 g, F	450 g, NND	Acardiac, SF
TCTA	35 + 2, CS	2300 g, F	1800 g, F	SF
DCTA	34 + 1, CS	1760 g, M	1940 g, M	SF
DCTA	36 + 6, CS	2495 g, F	IUD	IUD
DCTA	18, miscarriage	IUD	IUD	IUD
DCTA	22, TOP	IUD	IUD	IUD
MCTA	27 + 6, CS	IUD	NND	NND
MCTA	24, TOP	IUD	IUD	IUD
DCTA	33 + 2, CS	1540 g, F	1090 g, F	SF
DCTA	20 + 3, VD	IUD	IUD	IUD
TCTA	21 + 5, miscarriage	IUD	IUD	IUD
DCTA	24 + 5, miscarriage	NND	NND	NND
DCTA	20 + 6, TOP	IUD	IUD	IUD
MCDA	21 + 3, VD	IUD	IUD, cleft lip and palate	IUD, anencephalic, acardiac
MCTA	29 + 6, VD	1320 g, F, refused for NICU, NND	810 g, F, refused for NICU, NND	550 g, F, refused for NICU, NND
MCTA	27 + 6, VD	970 g, M	1220 g, F, refused for NICU, NND	1000 g, F, refused for NICU, NND
MCTA	24 + 3, miscarriage	IUD	IUD	IUD
DCTA	28 + 6, CS	660 g, F, refused for NICU, NND	870 g, F, refused for NICU, NND	880 g, F, refused for NICU, NND
DCTA	37 + 3, CS	2550 g, F	2000 g, M, brain damage	223 g, IUD

*CS* Cesarean section, *TOP* Termination of pregnancy, *VD* Vaginal delivery, *M* Male, *F* Female, *IUD* Intrauterine death, *SF* Selective feticide, *GA* Gestational age, *DCTA* Dichorionic triamniotic, *MCTA* Monochorionic triamniotic, *MCDA* Monochorionic diaminiotic, *DCDA* Dichorionic diaminiotic, *TCTA* Trichorionic triaminiotic, *NND* Neonatal death, *NICU* Neonatal intensive care unit

### Perinatal outcomes and birth weight discordance in triplet pregnancies without fetal loss

**Fig. 3:**
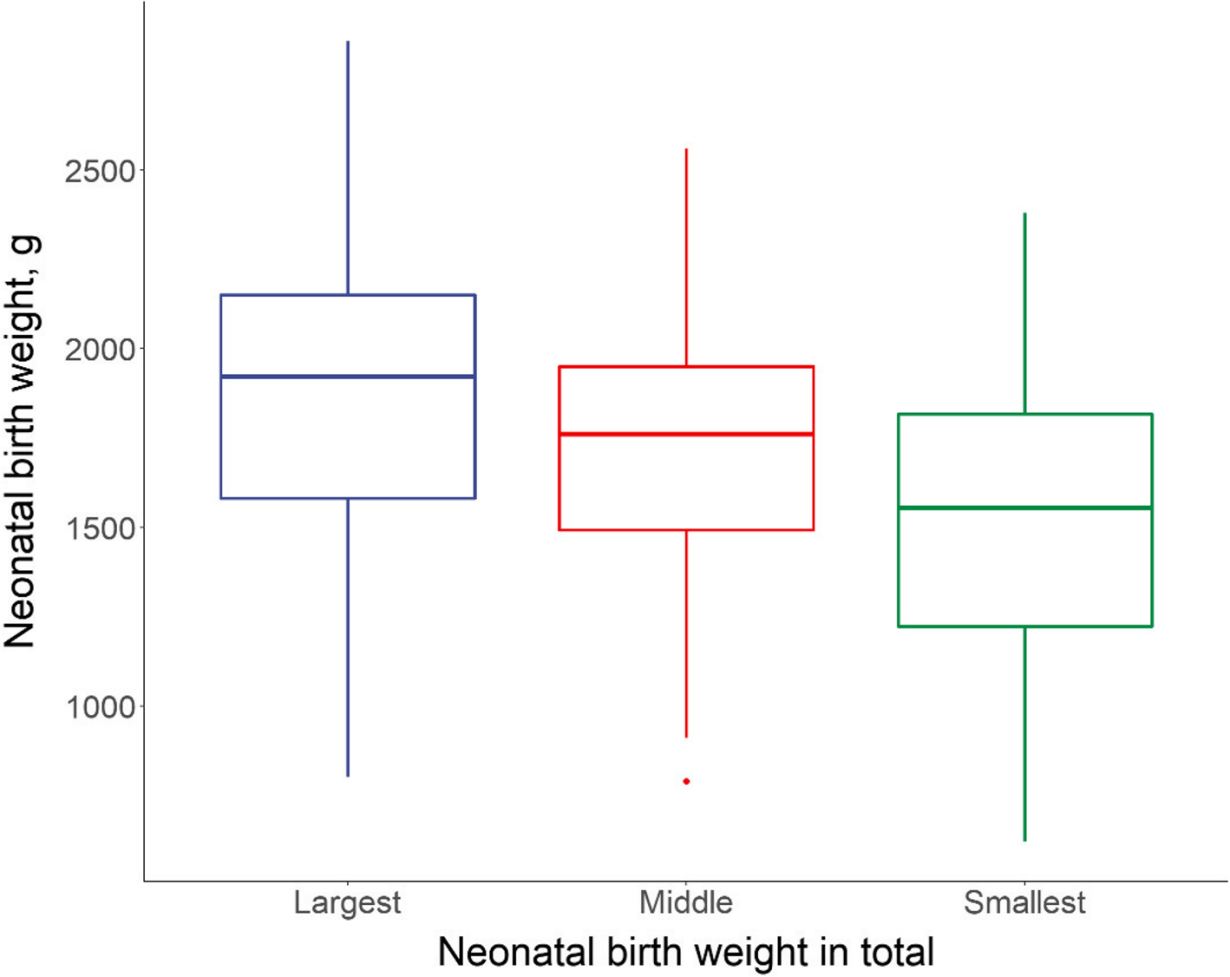
Birth weight of 99 triplet pregnancies

**Fig. 4:**
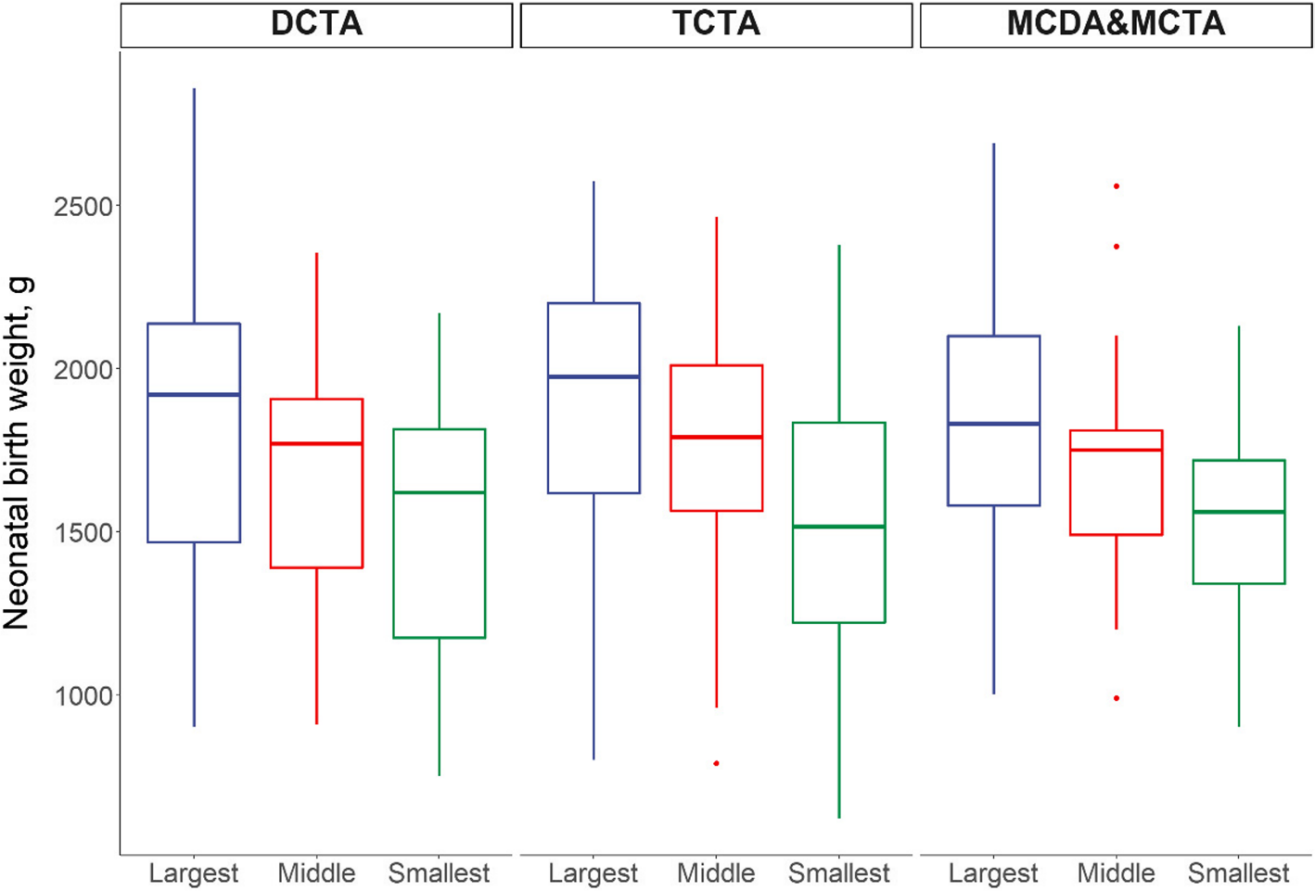
Birth weight of 99 triplet pregnancies based on chorionicity

Among the 99 triplet pregnancies, we compared the maternal demographic characteristics according to chorionicity and the mode of pregnancy, as well as pregnancy complications. Monochorionic triplet pregnancies showed a higher proportion of in vitro fertilization–embryo transfer (IVF-ET), while dichorionic pregnancies were primarily conceived naturally. In terms of pregnancy complications (*P* < 0.001), TC triplets showed a higher incidence of intrahepatic cholestasis during pregnancy (ICP), while monochorionic triplets presented a higher proportion of hypertension. These statistics are listed in Table 4, while the neonatal birth weights based on the chorionicity distribution of the 99 cases are depicted in Figs. 3 and 4.

**Table 4:** Maternal clinical characteristics of triplet pregnancies without fetal loss

	DCTA (41)	MCDA or MCTA (21)	TCTA (37)	*P*
Age (IQR)^a^	29 (26.50–32.00)	27 (26.00–32.50)	27.00 (23.50–33.50)	0.582
Pre-BMI (IQR)^a^	21.51 (19.34–23.03)	21.63 (19.49–22.32)	22.19 (19.93–25.17)	0.156
BMI-delivery (IQR)^a^	27.97 (26.23–29.92)	28.13 (27.16–30.96)	28.76 (27.26–31.84)	0.289
Gravidity (IQR)^a^	2 (1–3)	2 (1–3)	2 (1–3)	0.890
Parity (IQR)^a^	0 (0–1)	0 (0–0.5)	0 (0–0)	0.226
Mode of pregnancy, n (%)^b^				** < 0.001**
Natural	23 (56.01)	19 (90.48)	16 (43.24)	
Ovulation induction	3 (7.32)	0 (0)	9 (24.32)	
IVF-ET	15 (36.59)	2 (9.52)	12 (32.43)	
Pregnancy complication, n (%)^b^				** < 0.001**
Hypertension	4 (9.76)	7 (33.33)	7 (18.92)	
ICP	16 (39.92)	5 (23.81)	15 (40.54)	
GDM	16 (39.92)	3 (14.29)	5 (13.51)	

*pre-BMI* Body mass index before pregnancy, *BMI-delivery* Body mass index before delivery, *IVF-ET* In vitro fertilization–embryo transfer, *ICP* Intrahepatic cholestasis during pregnancy, *GDM* Gestational diabetes mellitus, *IQR* Interquartile range

^a^Described as the median and IQR, compared by Kruskal–Wallis H test

^b^Association test

The perinatal outcomes and BWD of the 99 triplet pregnancies with no fetal loss based on chorionicity were also collected and analyzed. The gestational age at birth varied significantly among different chorionicities (*P* < 0.001). Regarding the incidence of neonatal complications, neonatal hypoproteinemia (*P* < 0.001), hyperbilirubinemia (*P* < 0.019), and anemia (*P* < 0.003) varied significantly among different chorionicities, as did the rate of transfer to the NICU (*P* < 0.001), with the TCTA group showing the highest rate of 32.4%. Regarding treatment in the NICU, the rates of transfusion and CPAP were significantly higher in DCTA triplets (*P* = 0.019 and *P* = 0.01, respectively), while the duration of CPAP also differed significantly according to chorionicity (*P* = 0.012). Details of the neonatal outcomes based on chorionicity are shown in Table 5.

**Table 5:** Perinatal outcomes based on chorionicity

	DCTA (*n* = 123)	TCTA (*n* = 111)	MCDA or MCTA (*n* = 63)	*P*
GA at birth (%)^a^				** < 0.001**
~ 31 + 6	27 (22.0)	24 (21.62)	9 (14.29)	
32 ~ 33 + 6	45 (36.59)	27 (24.32)	21 (33.33)	
≥ 34	51 (41.46)	60 (54.05)	33 (52.38)	
Survival rate (%)^a^	116 (94.3)	108 (97.3)	60 (95.2)	0.529
Rate of transfer to NICU (%)^a^	20 (12.2)	36 (32.4)	13 (20.6)	** < 0.001**
Apgar score at 5 min (mean (SD))^b^	9.46 (1.00)	9.50 (1.44)	9.55 (0.97)	0.887
Malformation^a^	12 (9.8)	7 (6.3)	10 (15.9)	0.124
Asphyxia^a^	10 (8.1)	7 (6.3)	3 (4.8)	0.669
HIE^c^	2 (1.6)	0 (0.0)	2 (3.2)	0.206
RDS^a^	29 (23.6)	24 (21.6)	14 (22.2)	0.936
IVH^a^	28 (22.8)	26 (23.4)	18 (28.6)	0.66
Grade_of_IVH (%)^a^				0.097
Non	95 (77.2)	85 (76.6)	44 (69.8)	
Grade I	2 (1.6)	8 (7.2)	2 (3.2)	
Grade II	17 (13.8)	12 (10.8)	14 (22.2)	
Grade III	7 (5.7)	6 (5.4)	1 (1.6)	
Grade IV	2 (1.6)	0 (0.0)	2 (3.2)	
NEC (%)^c^	2 (1.6)	2 (1.8)	1 (1.6)	> 0.999
ROP (%)^a^	14 (11.7)	13 (11.9)	9 (14.5)	0.845
Neonatal_sepsis (%)^c^	6 (4.9)	2 (1.8)	1 (1.6)	0.374
Pneumonia (%)^a^	54 (43.9)	44 (39.6)	35 (55.6)	0.124
Apnea (%)^a^	27 (22.0)	15 (13.5)	8 (12.7)	0.139
Bronchopulmonary_dysplasia (%)^c^	7 (5.7)	3 (2.7)	1 (1.6)	0.346
CHD (%)^a^	47 (38.2)	29 (26.1)	16 (25.4)	0.076
Respiratory_failure (%)^a^	17 (13.8)	10 (9.0)	7 (11.1)	0.511
Hypoglycemia (%)^a^	22 (17.9)	19 (17.1)	10 (15.9)	0.942
Hypoproteinemia (%)^a^	14 (11.4)	39 (35.1)	18 (28.6)	** < 0.001**
Electrolyte_disturbance (%)^a^	21 (17.1)	23 (20.7)	16 (25.4)	0.403
Hyperbilirubinemia (%)^a^	57 (46.3)	41 (36.9)	16 (25.4)	**0.019**
Neonatal_scleredema (%)^c^	4 (3.3)	3 (2.7)	6 (9.5)	0.11
Brain_injury (%)^b^	7 (5.7)	2 (1.8)	3 (4.8)	0.296
Aerothorax (%)^c^	1 (0.8)	0 (0.0)	0 (0.0)	1
Pneumomediastinum (%)^c^	0 (0.0)	0 (0.0)	0 (0.0)	1
Gastrointestinal_bleeding (%)^a^	8 (6.5)	5 (4.5)	7 (11.1)	0.245
DIC (%)^c^	2 (1.6)	0 (0.0)	1 (1.6)	0.435
Pulmonary_hemorrhage (%)^c^	7 (5.7)	2 (1.8)	2 (3.2)	0.316
Natural_bleeding (%)^c^	0 (0.0)	0 (0.0)	1 (1.6)	0.212
Periventricular_leukomalaciain (%)^c^	0 (0.0)	3 (2.7)	2 (3.2)	0.099
Anemia (%)^a^	35 (28.5)	12 (10.8)	11 (17.5)	**0.003**
Therapy in NICU (%)^a^				
Blood_transfusions^a^	23 (18.7)	7 (6.3)	9 (14.3)	**0.019**
Sputum_bacterial_cultures^c^	120 (97.6)	107 (96.4)	63 (100.0)	0.384
Trachea_intubation^a^	12 (9.8)	6 (5.4)	6 (9.5)	0.425
Duration_of_trachea_intubation (mean (SD))^b^	0.57 (2.66)	0.38 (2.25)	0.97 (3.77)	0.406
CPAP^a^	43 (35.0)	23 (20.7)	11 (17.5)	**0.01**
Duration_of_CPAP (median [IQR])^d^	0.00 [0.00, 3.00]	0.00 [0.00, 0.00]	0.00 [0.00, 0.00]	**0.012**
Nasal_cannula^a^	28 (22.8)	16 (14.5)	14 (22.6)	0.234
Duration_of_nasal_cannula (mean (SD))^b^	1.79 (4.75)	1.21 (3.65)	1.48 (4.40)	0.584
Hood_oxygen (%)^a^	8 (6.5)	4 (3.6)	2 (3.2)	0.469
Duration_of_Hood_oxygen (mean (SD))^b^	0.42 (1.91)	0.19 (1.14)	0.10 (0.53)	0.277
BW_H (mean (SD))^b^	1853.05 (442.72)	1886.49 (427.34)	1867.81 (388.06)	0.835
BW_L (mean (SD))^b^	1517.61 (402.60)	1534.59 (429.20)	1536.95 (344.87)	0.931
BW_M (mean (SD))^b^	1686.95 (363.47)	1749.86 (396.60)	1703.29 (370.38)	0.454
BW_Discordance (mean (SD))^b^	0.17 (0.14)	0.18 (0.14)	0.17 (0.11)	0.845
BW_Discordance (%)^a^				** < 0.001**
< 15%	69 (56.1)	57 (51.4)	30 (47.6)	
15%–20%	15 (12.2)	21 (18.9)	12 (19.0)	
20%–25%	15 (12.2)	6 (5.4)	18 (28.6)	
> 25%	24 (19.5)	27 (24.3)	3 (4.8)	

*GA* Gestational age, *BW* Birth weight, *HIE* Hypoxic ischemic encephalopathy, *RDS* Respiratory distress syndrome, *IVH* Intraventricular hemorrhage, *NEC* Necrotizing enteritis, *ROP* Retinopathy of prematurity, *CHD* Congenital heart disease, *DIC* Disseminated intravascular coagulation, *CPAP* Continuous positive airway pressure, *BW_H* Largest fetus, *BW_L* Smallest fetus, *BW_M* Middle fetus, *SD*: Standard deviation, *NICU* Neonatal intensive care unit, *IQR* Interquartile range

^a^Association test

^b^One-way ANOVA

^c^Fisher’s exact test

^d^Kruskal–Wallis *H* Test

### Birth weight discordance and its effect on perinatal outcome based on chorionicity

We first analyzed the average BW according to chorionicity and found no significant difference. Then, we compared the BW between the largest and the smallest neonates based on different chorionicities, which did show a significant difference (*P* < 0.001). The collected statistics indicated that up to 50% of DCTA (56.1%) and TCTA (51.4%) triplets showed a BWD < 15%, while this was not observed in monochorionic triplets (47.6%). Other neonatal complications, including hypoxic-ischemic encephalopathy (HIE), necrotizing enteritis (NEC), and congenital heart disease (CHD), were not significantly different among different types of triplets. The comparison is presented in Table 5.

The multivariate binary logistic regression analysis results are shown in Table 6. TCTA pregnancy decreased the risk of neonatal anemia (adjusted odds ratio [AOR] = 0.084 (*P* = 0.002, 95% CI: 0.018–0.395)) and the need for blood transfusion after birth (AOR = 0.119 (*P* = 0.011, 95% CI: 0.023–0.617)), while a BWD > 25% increased the risk of neonatal anemia (AOR = 10.135 (*P* = 0.008, 95% CI: 1.853–55.447)) and the need for blood transfusion after birth (AOR = 7.127 (*P* = 0.021, 95% CI: 1.339–37.934)). Meanwhile, TC pregnancy, MCDA or MCTA, and BWD > 25% all increased the risk of neonatal hypoproteinemia, with AORs of 4.629 (0.008, 1.504–14.243), 5.123 (0.011, 1.463–17.942) and 5.343 (0.043,1.056–27.035), respectively.

**Table 6:** Multivariate binary logistic regression analysis on the effect of BWD on perinatal outcome

Perinatal outcomes OR	TCTA		MCDA or MCTA		BWD > 25%	
	**AOR (95% CI)**	***P*-value**	**AOR (95% CI)**	***P*-value**	**AOR (95% CI)**	***P*-value**
Neonatal anemia	0.084 (0.018–0.395)	0.002	0.436 (0.100–1.901)	0.269	10.135 (1.853–55.447)	0.008
Blood transfusion therapy for neonates	0.119 (0.023–0.617)	0.011	0.922(0.161–5.265)	0.927	7.127 (1.339–37.934)	0.021
Hypoproteinemia	4.629 (1.504–14.243)	0.008	5.123 (1.463–17.942)	0.011	5.343 (1.056–27.035)	0.043
Respiratory failure	0.265 (0.053–1.32)	0.105	1.459 (0.261–8.136)	0.667	2.558 (0.735–8.899)	0.14
CHD	0.474 (0.163–1.381)	0.171	0.601 (0.177–2.042)	0.415	0.451 (0.124–1.639)	0.227
Hyperbilirubinemia	0.491 (0.173–1.392)	0.181	0.376 (0.110–1.287)	0.119	2.465 (0.448–13.567)	0.300
CPAP	0.524 (0.169–1.63)	0.265	0.41 (0.106–1.577)	0.194	1.882 (0.556–6.369)	0.309
Apnea	0.539 (0.181–1.602)	0.266	0.908 (0.261–3.161)	0.879	1.894 (0.518–6.922)	0.334
Pneumonia	0.542 (0.182–1.616)	0.272	1.011 (0.290–3.529)	0.986	1.839 (0.531–6.362)	0.336
Malformation	0.72 (0.219–2.366)	0.588	1.414 (0.396–5.043)	0.594	0.629 (0.178–2.225)	0.472
IVH	1.279 (0.441–3.711)	0.650	2.373 (0.708–7.953)	0.161	1.486 (0.470–4.695)	0.500
RDS	0.821 (0.258–2.607)	0.738	0.564 (0.138–2.307)	0.425	0.837 (0.222–3.154)	0.792
Asphyxia	1.222 (0.234–6.381)	0.812	1.281 (0.188–8.734)	0.800	1.111 (0.295–4.190)	0.876
Electrolyte disturbance	0.912 (0.326–2.552)	0.860	1.398 (0.426–4.587)	0.580	0.924 (0.247–3.451)	0.907
Hypoglycemia	1.078 (0.396–2.935)	0.884	0.637 (0.181–2.243)	0.483	0.976 (0.308–3.091)	0.966

The reference category of TCTA and MCDA or MCTA is DCTA, while that for BWD > 25 is BWD < 25%

*OR* Odds ratio, *AOR* Adjusted odds ratio, *CI* Confidence interval, *CHD* Congenital heart disease, *CPAD* Continuous positive airway pressure, *IVH* Intraventricular hemorrhage, *RDS* Respiratory distress syndrome

## Discussion

We reviewed a total of 136 triplet pregnancies at our tertiary hospital between January 1, 2001, and December 31, 2021. The distribution of BWD was found to differ significantly according to different chorionicities (*P* < 0.001): More than half of the cases in the DC and TC groups had a BWD < 15%, while nearly half of those in the MC group had a BWD < 50% (47.6%). TC pregnancy decreased the risk of neonatal anemia (OR = 0.084) and the need for blood transfusion therapy after birth (OR = 0.119). In contrast, BWD > 25% increased the risk of neonatal anemia (OR = 10.136) and the need for blood transfusion after birth (OR = 7.127). TC pregnancy, MCDA or MCTA, and BWD > 25% increased the risk of neonatal hypoproteinemia, with AORs of 4.629, 5.123, and 5.343, respectively.

The effect of the fetal number independent of chorionicity is demonstrated by the lower BW of TC triplets compared to that of dichorionic twins [17]. DCTA/MCTA triplets demonstrated greater BWD than TCTA triplets (*P* = 0.049). Moreover, the rate of severe BWD (> 35%) was 2.5-fold higher in DCTA/MCTA compared to that in TCTA pregnancies (26.1% vs. 10.4%), although this difference was not significant [14].

BWD is common in live-born twins and is associated with perinatal outcomes [18–22]. Approximately 20% of triplet sets experience some level of discordance, and severe discordance (> or = 35%) occurs in approximately 10% of triplet sets [20]. In this study, TCTA was demonstrated to be a protective factor that prevents triplet neonates from developing anemia and requiring transfusion therapy after birth. At the same time, BWD > 25% was determined to be a risk factor. Additionally, both TCTA and BWD > 15% were found to increase the risk of neonatal hypoproteinemia. Based on our clinical observations, this finding could be explained by differences in early embryonic angiogenesis among different chorionic triplets. However, further investigations into the developmental and behavioral abnormalities of these neonates will help us to better understand this phenomenon.

Acardiac anomaly is a unique complication of monochorionic twinning that has a reported incidence of 1/35000 pregnancies. The occurrence of an acardiac fetus in a triplet pregnancy is extremely rare [23]. In our study, the total incidence of the acardiac anomaly was 5.42 per 100,000 (9/165985). Monoamniotic twin pregnancies have a high incidence of fetal anomalies (15–25%), but the presence of a conjoined twin in monoamniotic twin pregnancies is even rarer, with an incidence of only 1.5 per 100,000 pregnancies [24]. Two conjoined twins with DCDA chorionicity were reported in our study, with an incidence of 1.20 per 100,000, which is lower than that reported previously.

Selective feticide was implemented in 11 cases in our study, including seven and four cases of reduction to twin and singleton pregnancies, respectively. The mean birth weeks and BWs in cases reduced to twin pregnancies (7/11) were 33.65 weeks and 1731.43 g, while those in cases reduced to singleton pregnancies (4/11) were 37.50 weeks and 3045.00 g. This outcome is not uncommon. A study of 285 trichorionic and dichorionic triplet pregnancies with abdominal fetal reduction at 11–14 weeks demonstrated that triplet pregnancies reduced to twin pregnancies had a higher rate of preterm birth compared to those reduced to singletons pregnancies [25]. Notably, Hessami et al. [26] suggested that a reduction to a twin pregnancy resulted in a significantly lower gestation age at birth (weeks) than a reduction to a singleton pregnancy (95% CI: − 2.80– − 1.61; *P* < 0.001). Furthermore, a reduction in twin pregnancies was related to lower BW and a higher risk of preterm birth, both at < 32 and < 34 weeks of gestation. Despite our study including a smaller sample size, our findings are consistent with those reported in these studies.

Previous studies have shown that gestational diabetes is more common in triplet pregnancies than in single pregnancies, with a relative risk of 1.5 [27]. Moreover, a previously published study reported that twin pregnancies had a higher prevalence of ICP (20.9%) than singleton pregnancies (4.7%) [28]. Studies have also highlighted that hypertensive disorders of pregnancy (HDP) are proportional to the number of fetuses: singletons, 6.5%; twins, 12.7%; and triplets, 20.0% [29]. Data have suggested that participants with triplet pregnancies exhibited higher blood pressure and reduced maternal cardiovascular function compared to those with single or twin pregnancies [30]. The rates of GDM, ICP, and HDP in this study were as high as 24.2% (24/99), 36.3% (36/99), and 18.10% (18/99), respectively. Furthermore, TC triplets with a higher incidence of ICP and monochorionic presented with a higher proportion of hypertension, which is in agreement with the results of previously published studies.

A previous study reported triplet pregnancies with a mean BW and birth gestational age of 1680 g and 31.7 weeks and found that more than 60% of triplets gave birth before 34 weeks and almost 100% before 37 weeks, with an infant mortality rate of up to 52.5% [6]. It has also been reported that the mean gestational age at delivery of triplets is 32.3 ± 3.6 weeks and the average BW is 1,726 ± 500 g [7]. In this study, 51.51% of triplets were born before 34 weeks and all 99 cases were born before 37 gestational weeks, with a mean delivery age of 33.3 ± 2.2 weeks. The average BW was 1426.1 ± 427.5 g. The National Institute for Health and Care Excellence (NICE) has suggested that the timing of birth should be individualized and tailored to the woman’s needs and circumstances in triplet pregnancies, with a shared chorion or amnion [31]. In this retrospective study, the birth age was significantly differently distributed among different chorionic triplets.

Previous studies have highlighted that the risks of admission to the NICU, respiratory distress, sepsis, necrotizing enterocolitis, and perinatal and intrauterine mortality are higher in non-TCTA pregnancies than in TCTA pregnancies [1]. The rate of transfer to the NICU was also significantly different among the three groups (*P* < 0.001), with the TCTA group showing the highest rate of 32.4%. Neonatal complications such as hypoproteinemia, hyperbilirubinemia, and anemia also differed significantly after analysis (*P* < 0.005). The rates of transfusion and CPAP were significantly higher in DCTA triplets (*P* < 0.005), while the duration of CPAP also differed among different chorionicities (*P* = 0.012). Our findings did not entirely align with prior research, as there were fewer non-TCTA pregnancies than TCTA pregnancies.

The total occurrence of triplet pregnancy in our hospital was 0.094% (156/165985), which was inconsistent with the results of a previous investigation [1]. Monochorionic triplet pregnancies occur at a rate of approximately 1 in 45,500 deliveries [32]. An analysis of a large sample of clinical data revealed that the rate of DCTA has increased due to the increased performance of ART [11]. Stratulat et al. classified triplet pregnancies as 67.3% TCTA, 25.4% DCTA, 1.8% MCTA, and 5.4% DCDA of cases according to the upsilon (Y) zone [2]. In this study, 28.6% were TCTA, 4.4% were MCDA, 4.4% were DCDA, 43.3% were DCTA, and 19.1% were MCTA. The total occurrence rate of monochorionic triplet pregnancies in our study was 1.93 in 10,000 pregnancies, which is higher than that reported in previous studies; this difference was potentially due to the characteristics of the triplet pregnancies transferred to our hospital, the tertiary referral center of West China. We also believe that several other factors contribute to this difference, including variations in the period of statistical cases, advancements in prenatal diagnosis and assisted reproductive technology, and discrepancies in chorionicity confirmation.

A meta-analysis of nine studies revealed a miscarriage rate of 4.83% before 22 weeks, including 5% in IUD, 4.42% NND, and 1.73% PND [1]. Dudenhausen et al., in their prospective population-based study of extremely preterm births between 22 + 0 and 31 + 6 weeks of gestation in 19 regions from 11 European countries, reported that 28.9% of mothers with a triplet pregnancy experienced at least one neonatal death [33]. The mortality rate (fetal death at > 22 weeks of gestation; neonatal death) in triplets was 2.6% and included 2.1% of TT triplet pregnancies, 3.2% of DT triplet pregnancies, and 5.3% of MT triplet pregnancies [34]. The rates of IUD, NND, and PND in this study were 10.29% (42/408), 13.07% (57/408), and 24.26%, respectively, in 136 triplet pregnancies. Notably, in this study, IUD or NND mostly occurred in pregnancies with fetal loss or cases requiring intrauterine intervention. Considering the different methods of grouping, the outcomes in this study are not fully generalizable to those of previous studies.

Given our clinical experience, we hypothesize that variations in early embryonic angiogenesis among different chorionic triplets result in diverse intrauterine growth and development patterns. Neonatal weight plays a pivotal role in determining postnatal clinical interventions, leading to varied clinical outcomes. We suspect that this falls under the umbrella of fetal diseases, necessitating further investigation.

### Strengths and limitations

The primary strengths of this study lie in the comparison of maternal and neonatal outcomes according to chorionicity at a single tertiary hospital. Furthermore, we categorized the BWD according to triplet pregnancies of varying chorionicities.

However, it is important to note that this was a retrospective cohort study, and the number of cases with different chorionicities were relatively limited. Furthermore, the neonatal outcome data were limited to from birth until discharge. Therefore, the results and conclusions necessitate a larger sample size and robust and long-term follow-up statistical analyses.

## Conclusion

In this study, we observed BWD according to chorionicity and its influence on perinatal outcomes. The BWD differed significantly according to chorionicity. Additionally, TC pregnancies reduced the risk of neonatal anemia and need for blood transfusion, but increased the risk of neonatal hypoproteinemia. In contrast, the BWD between the largest and smallest triplets increased the risk of neonatal anemia and the need for blood transfusion. TC pregnancy, MCDA or MCTA, and BWD > 25% increased the risks of neonatal hypoproteinemia. To date, the true discordance and its stratification have been rarely investigated owing to the lack of triplet pregnancies.

## Data Availability

The datasets used and/or analyzed during the current study are available from the corresponding author upon reasonable request.

## Abbreviations

ART: Assisted reproductive technology
DCTA: Dichorionic triamniotic
MCTA: Monochorionic triamniotic
TCTA: Trichorionic triamniotic
DCDA: Dichorionic diamniotic (containing monochorionic twin set)
MCDA: Monochorionic diamniotic
HIE: Hypoxic-ischemic encephalopathy
NEC: Necrotizing enteritis
CHD: Congenital heart disease
BWD: Birth weight discordance
IUD: Intrauterine death
NND: Neonatal death
PND: Perinatal death
NICU: Neonatal intensive care unit
IQR: Interquartile range
IVF-ET: In vitro fertilization–embryo transfer
ICP: Intrahepatic cholestasis during pregnancy
BMI: Body mass index
HGB: Hemoglobin
AOR: Adjusted odds ratio
CI: Confidence interval
NICE: The National Institute for Health and Care Excellence
